# No-Reference Image Quality Assessment Based on Dual-Domain Feature Fusion

**DOI:** 10.3390/e22030344

**Published:** 2020-03-17

**Authors:** Yueli Cui

**Affiliations:** School of Electronic and Information Engineering, Taizhou University, Taizhou 318017, China; cuiyueli@tzc.edu.cn

**Keywords:** no-reference image quality assessment, dual-domain feature fusion, curvelet transform, image entropy

## Abstract

Image quality assessment (IQA) aims to devise computational models to evaluate image quality in a perceptually consistent manner. In this paper, a novel no-reference image quality assessment model based on dual-domain feature fusion is proposed, dubbed as DFF-IQA. Firstly, in the spatial domain, several features about weighted local binary pattern, naturalness and spatial entropy are extracted, where the naturalness features are represented by fitting parameters of the generalized Gaussian distribution. Secondly, in the frequency domain, the features of spectral entropy, oriented energy distribution, and fitting parameters of asymmetrical generalized Gaussian distribution are extracted. Thirdly, the features extracted in the dual-domain are fused to form the quality-aware feature vector. Finally, quality regression process by random forest is conducted to build the relationship between image features and quality score, yielding a measure of image quality. The resulting algorithm is tested on the LIVE database and compared with competing IQA models. Experimental results on the LIVE database indicate that the proposed DFF-IQA method is more consistent with the human visual system than other competing IQA methods.

## 1. Introduction

Many image processing tasks (e.g., image acquisition, compression, transmission, restoration, etc.) often cause different types of distortion at different levels, so perceived quality assessment has been receiving more and more attention [1]. It can be divided into subjective IQA and objective IQA. The conventional way of measuring image quality is to solicit the opinion of human observers. However, such subjective IQA methods are cumbersome and time-consuming, so they are difficult to be incorporated into automatic systems. Therefore, objective IQA methods have more actual significance in practical applications [2,3].

Depending on whether there are available reference images, objective IQA methods can be divided into three categories: full-reference IQA (FR-IQA) [4,5,6,7,8,9,10,11,12,13,14,15,16,17], reduced-reference IQA (RR-IQA) [18,19] and no-reference IQA (NR-IQA) [20,21,22,23,24,25,26,27,28,29,30,31]. FR-IQA and RR-IQA require the provision of multiple reference images and partial information of them, respectively. In most cases, the reference image is not always available, so the NR-IQA are the unique ones to be embedded into the actual application system.

According to the scope of application, the current methods of NR-IQA can be roughly divided into two categories: special methods for specific types of distortion [20,21,22] and general methods for various types of distortion [23,24,25,26,27,28,29,30,31]. Considering that special-purpose algorithms need to acquire the type of distortion such as blur, noise, compression, etc., their scope of application is limited. Therefore, research on general-purpose methods has become a hot topic in the field of IQA, including two-stage framework models and global framework models.

At present, BIQI [23], DIIVINE [24], SSEQ [25], and CurveletQA [26] are the representative methods of two-stage framework IQA models. BIQI extracts statistical features from the wavelet coefficients and utilizes distortion classification to judge the specific type of distortion, and then adopts the corresponding regression model to evaluate the image. On the basis of BIQI, DIIVINE obtains sub-band coefficients with different directions by the multi-scale wavelet decomposition and extracts several statistical features to predict the image quality by employing support vector machine (SVM) regression. SSEQ combines local spatial and spectral entropy features extracted from the distorted images after distortion classification. CurveletQA proposes a two-stage framework of distortion classification followed by quality assessment, and a set of statistical features are extracted from a computed image curvelet representation.

Representative global framework of NR-IQA models include BLIINDS-I [27], BLIINDS-II [28], GRNN [29], BRISQUE [30], and NIQE [31]. BLIINDS-I combined features of contrast, sharpness and anisotropy in discrete cosine transform (DCT) domain by a down-sampling operation on the image and adopted a probability model to predict image quality. BLIINDS-II predicted quality score by using statistical features extracted from local image blocks on the basis of BLIINDS-I. GRNN extracted complementary perceptual features of phase consistency, gradient and entropy, and adopted a generalized regression neural network to establish a mapping between visual features and subjective scores to predict image quality. BRISQUE investigated the statistical rules of images from the perspective of the spatial domain and utilized support vector regression (SVR) to establish a mapping between statistical features and mean opinion score (MOS). NIQE evaluated image quality by measuring the distance between the statistical features of distorted images and natural images. In view of visual perception and statistical characteristics, this paper proposes a novel NR-IQA algorithm based on dual-domain feature fusion, dubbed as DFF-IQA. In the spatial domain, features of weighted local binarization pattern (WLBP), naturalness, and spatial entropy are extracted. In the frequency domain, asymmetrical generalized gaussian distribution (AGGD) fitting parameters of curvelet coefficients, oriented energy distribution (OED), and spectral entropy features are extracted. The features extracted from the dual-domain are fused to form a quality-aware feature vector, and then random forest regression is employed to predict the image quality score. At last, we validate the performance of the proposed DFF-IQA method on the LIVE database. In summary, the main contributions of this work are:

(1) We analyze and extract the perceptual features in dual-domain, and the fused quality-aware feature vector has been verified to promote the performance of quality evaluation.
(2) We compare the representative FR/NR-IQA models with our DFF-IQA model. The experimental results show that the proposed method has better performance and has good consistency with human subjective perception.

The remainder of this paper is organized as follows. In Section 2, the proposed model is presented. In Section 3, we illustrate and discuss the experimental results. Finally, we conclude our paper in Section 4.

## 2. Proposed DFF-IQA Method

**Figure 1:**
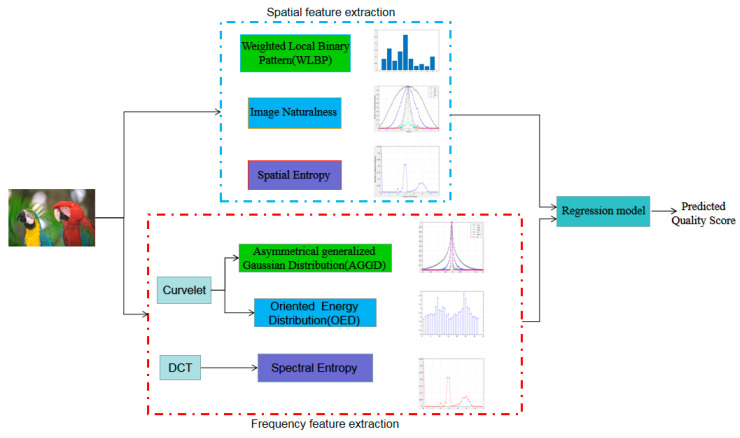
Overview of the proposed DFF-IQA framework.

As shown in Figure 1, this paper proposes a novel NR-IQA algorithm based on dual-domain feature fusion, dubbed as DFF-IQA. Firstly, in the spatial domain, we extract several features about weighted local binary pattern (WLBP) [32], naturalness and entropy from the distorted image. WLBP describes the texture characteristic of distorted images by adopting the statistical features of gradient-weighted LBP histogram, and the naturalness feature is represented by the fitting parameters of the generalized gaussian distribution (GDD). Spatial entropy is computed based on image blocks. Secondly, in the frequency domain, the DCT and curvelet transforms are implemented on distorted image based on the blocks respectively. In DCT domain, spectral entropy feature is extracted from image blocks. In curvelet transform domain, an asymmetric generalized Gaussian distribution (AGGD) model is employed to summarize the distribution of curvelet coefficients of the distorted image. Meanwhile, oriented energy distribution (OED) feature is further extracted to describe the curvelet coefficient. Thirdly, the features extracted in the dual-domain are fused to build the quality-aware feature vector. Finally, quality regression process by random forest is conducted to build the relationship between image features and quality score, yielding a measure of image quality. The framework of the proposed DFF-IQA algorithm is depicted in Figure 1.

### 2.1. Feature Extraction

#### 2.1.1. Weighted Local Binary Pattern (WLBP)

Local binary pattern (LBP) is an operator used to describe local texture features of images effectively and has shown good performance for evaluation of IQA tasks [33]. In this paper, the LBP coding with rotation invariance equivalent mode is employed, and gradient magnitude is adopted for weighting. Here, the gradient magnitude of the image ($G_{I}$) is obtained using the Prewitt filter. The calculation process is as follows:

(1) $$G_{I}=\sqrt{{\left(I*P_{h}\right)}^{2}+{\left(I*P_{v}\right)}^{2}}$$

where I is the input image, *P_h_* and *P_v_* are the Prewitt filters in the horizontal and vertical directions respectively, “*” represents the convolution operation, and $G_{I}$ is the gradient magnitude image of I.

We calculate the local rotation invariant uniform LBP operator $L_{P,R}^{}$ by:

(2) $$L_{P,R}^{}=\left\{ \begin{array}{ll} \sum_{i=0}^{P-1}z(G_{i}-G_{c}), & \psi (L_{P,R})\leq 2\\ P+1, & else \end{array}\right.$$

where *R* is the radius value, and *P* represents the number of neighboring points. $G_{c}$ indicates a center pixel at the position $\left(x_{c},y_{c}\right)$ in the corresponding images, and $G_{i}$ is a neighboring pixel (*x_p_*, *y_p_*) surrounding $G_{c}$:

(3) $$x_{p}=x_{c}+R\cos(2\pi \frac{p}{P})\;\mathrm{and}\;y_{p}=y_{c}-R\sin(2\pi \frac{p}{P})$$

where p∈{1, 2, …P} is the number of neighboring pixels sampled by a distance R from $G_{c}$ to $G_{i}$. In this case, *z*(*θ*) is the step function and defined by:

(4) $$z(\theta )=\left\{ \begin{array}{ll} 1, & \theta \geq T\\ 0, & \mathrm{otherwise} \end{array}\right.$$

where T indicates the threshold value. In addition, ψ(·) is used to compute the number of bitwise transitions:

(5) $$\begin{array}{cc} \psi (L_{P,R})= & \left\|z(G_{P-1}-G_{c})-z(G_{0}-G_{c})\right\|\\ & +\sum_{i=0}^{P-1}\left\|z(G_{i}-G_{c})-z(G_{i-1}+G_{c})\right\| \end{array}$$

where $L_{P,R}$ is the rotation-invariant operator:

(6) $$L_{P,R}=\min\left\{ROR(\sum_{p=1}^{P}z(G_{i}-G_{c})2^{p},k)\right\}$$

where k∈{1, 2, …,P}, and ROR(β,k) is the circular bit-wise right shift operator that shifts the tuple β by k positions. Finally, we obtain $L_{P,R}^{}$ with a length of P+2.

Prewitt filters with horizontal, vertical, main diagonal, and secondary diagonal directions were used to obtain the four gradient images in different directions by convolution operation. They are defined as $O_{d}=I*P_{d},\;d=1,2,3,4$, where $P_{d}$ represents the Prewitt filter with four different directions, I denotes the input image, and $O_{d}$ represents the gradient of the four directions. In this work, we use the maximum gradient magnitude *O*(*i*, *j*) calculated by Equation (7) as the LBP weight of each pixel:

(7) $$O(i,j)=Max(\left|O_{d}(i,j)\right|),d=1,2,3,4$$

where $\left|O_{d}(i,j)\right|$ represents the values of the gradient in four directions of (*i*, *j*) pixel point. Then the final weight map is obtained. The gradient magnitudes of pixels with the same WLBP pattern are accumulated $O_{H}(c)$, which can be regarded as the gradient-weighted WLBP histogram.

(8) $$O_{H}(c)=\sum_{i=1}^{w}\sum_{j=1}^{h}O(i,j)g(L_{P,R}^{}(i,j),c)$$

(9) $$g(x,y)=\left\{ \begin{array}{ll} 1, & x=y\\ 0, & else \end{array}\right.$$

where O(i,j) represents the maximum directional gradient response, that is, the weight map;w and h represent the length and width of the image, respectively; and c∈[0,C] is the LBP encoding patterns. In this paper, LBP of rotation invariant equivalence mode is used. At last we extract 10-dimensional statistical characteristics from pattern 1 to pattern 10 at a single scale.

**Figure 2:**
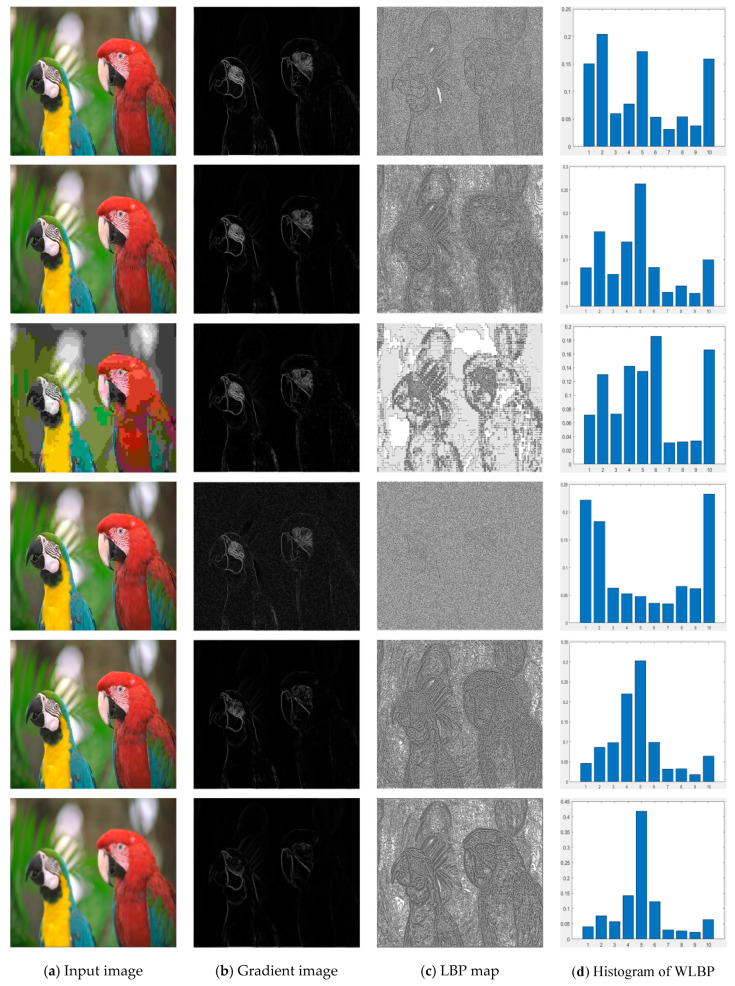
Pristine natural image and five distorted versions of it from the LIVE IQA database (“parrots” in LIVE database); from left column to right column are the input image, gradient image, LBP map, and histogram of WLBP. From top to bottom of the first column are pristine image with DMOS = 0, JPEG2000 compressed image with DMOS = 45.8920, JPEG compressed image with DMOS = 46.8606, white noise image with DMOS = 47.0386, fast-fading distorted image with DMOS = 44.0640, and Gaussian blur image with DMOS = 49.1911.

As shown in Figure 2d, we can find a significant difference in the WLBP distribution for different distortion types. The abscissa of the histogram is LBP coding pattern from pattern 1 to pattern 10. In the histogram of the pristine natural image, most number of the patterns are pattern 1, pattern 2, pattern 5, and pattern 10, and the other patterns are relatively few. When the images are distorted, the pattern distribution changes significantly. Therefore, the spatial distortion of the image can be described by extracting the WLBP feature of the image.

#### 2.1.2. Naturalness Feature

The locally mean subtracted contrast normalized (MSCN) coefficients have been successfully applied to measure their naturalness [30]. For each distorted image, its MSCN coefficients can be calculated by:

(10) $$M_{I}(i,j)=\frac{I(i,j)-\mu (i,j)}{\sigma (i,j)+C_{1}}$$

where I is the input image, $C_{1}$ is a constant that prevents instabilities from occurring when denominator tends to zero, and μ and σ are the mean and standard deviation of the distorted image, respectively. The calculation formulas are shown in Equations (11) and (12).

(11) $$\mu (i,j)=\sum_{k=-K}^{K}\sum_{l=-L}^{L}\omega_{k,l}E_{k,l}^{1}(i,j)$$

(12) $$\sigma (i,j)=\sqrt{\sum_{k=-K}^{K}\sum_{l=-L}^{L}\omega_{k.l}{\left(E_{k,l}^{1}(i,j)-\mu_{k,l}(i,j)\right)}^{2}}$$

where $\omega =\left\{\omega_{k,l}|k=-K,\ldots ,K,\;l=-L,\ldots ,L\right\}$ represents a 2D circularly-symmetric Gaussian weighting function sampled out to three standard deviations and rescaled to unit volume. In our implementation, we set K=L=3.

**Figure 3:**
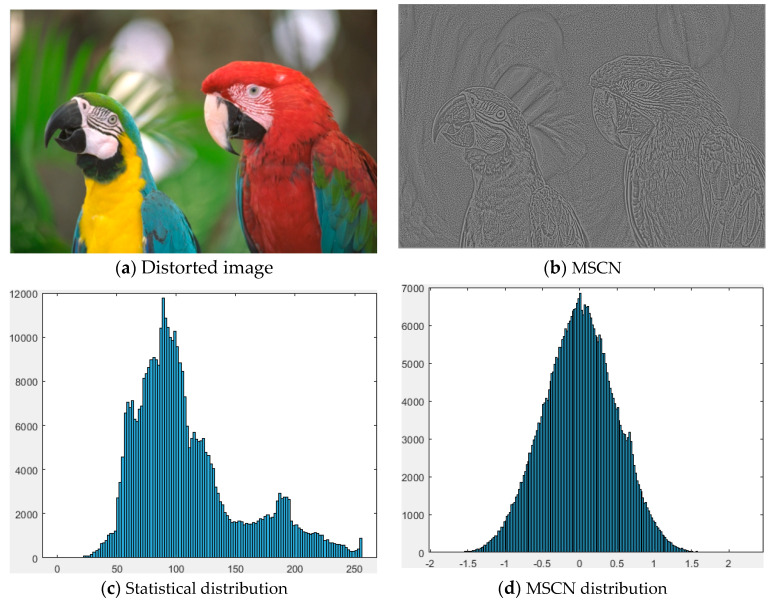
(**a**) Distorted image (“parrots” in LIVE database, type of JP2K compressed image with DMOS = 45.8920); (**b**) Corresponding MSCN coefficients image of (**a**); (**c**) Statistical distribution of the distorted image; (**d**) MSCN histogram distribution of MSCN coefficients.

Figure 3a,b shows the distorted image of JP2K and the corresponding MSCN coefficients, respectively; Figure 3c,d shows statistical distribution of the distorted image and MSCN histogram distribution of MSCN coefficients, respectively. As shown in Figure 3c,d, the distribution of MSCN coefficients is significantly different from the statistical distribution of the distorted image and approximates the Gaussian distribution. Therefore, the distribution of MSCN coefficient can be fitted by GGD to represent the degree of naturalness [30].

**Figure 4:**
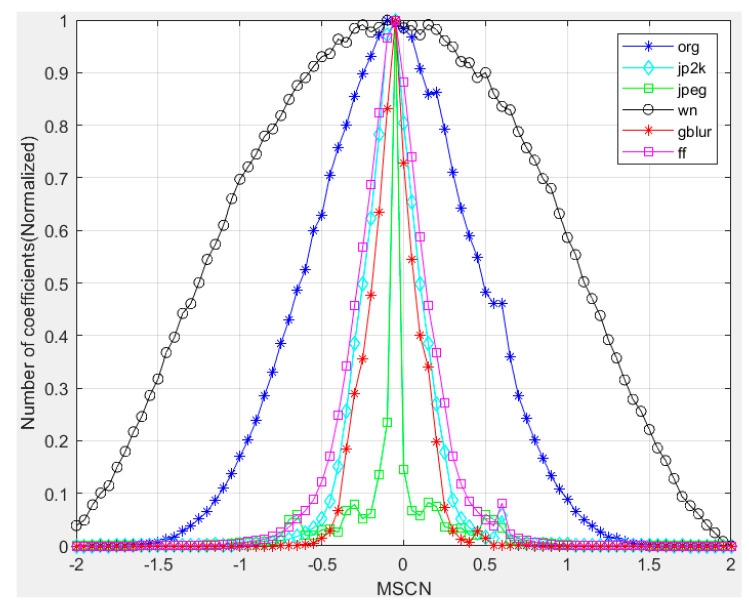
Histogram of MSCN coefficients for a reference image and its various distorted versions. Distortions from the LIVE IQA database. org: original image (i.e., Pristine natural image). jp2k: JPEG2000. jpeg: JPEG compression. wn: additive white Gaussian noise. blur: Gaussian blur. ff: Rayleigh fast-fading channel simulation.

Figure 4 plots a histogram of MSCN coefficients for a pristine natural image and for various distorted versions of it. Notice how the pristine image exhibits a Gaussian-like appearance, while each distortion modifies the statistics in its own characteristic way. For example, blur creates a more Laplacian appearance, while white-noise distortion appears to reduce the weight of the tail of the histogram. We have found that a generalized Gaussian distribution (GGD) can be used to effectively capture a broader spectrum of distorted image statistics, which often exhibit changes in the tail behaviour (i.e., kurtosis) of the empirical coefficient distributions where the GGD with zero mean is given by:

(13) $$f(x;\alpha ,\sigma^{2})=\frac{\alpha }{2\beta \Gamma (\frac{1}{\alpha })}\exp(-{\left(\frac{\left|x\right|}{\beta }\right)}^{\alpha })$$

where

(14) $$\beta =\sigma \sqrt{\frac{\Gamma (\frac{1}{\alpha })}{\Gamma (\frac{3}{\alpha })}}$$

where Γ(⋅) is the gamma function:

(15) $$\Gamma (a)=\int_{0}^{\infty }t^{a-1}e^{-t}dt\;a>0$$

#### 2.1.3. Local Spatial Entropy and Spectral Entropy

Although global entropy can reflect the overall information in the image, it cannot reflect the details in the image. Therefore, this paper uses entropies computed from local image blocks, on both the block spatial scale responses and also on the block DCT coefficients [25].

The spatial entropy is computed by:

(16) $$E_{s}=-\sum_{v}p(v)\log_{2}(p(v))$$

where v are the pixel values in a local block, with empirical probability density p(v).

The block DCT coefficient matrix $M_{C}$ is firstly computed on 8 × 8 blocks. Implementing of the DCT rather than the DFT reduces block edge energy in the transform coefficients. DCT coefficients are normalized by the following equation:

(17) $$P((i,j))=M_{C}{\left(i,j\right)}^{2}/{\sum_{i}\sum_{j}M_{C}(i,j)}^{2}$$

where 1≤i≤8, 1≤j≤8, and i,j≠1 (DC is excluded). Then the local spectral map could be computed by:

(18) $$E_{f}=-\sum_{i}\sum_{j}P(i,j)\log_{2}P(i,j)$$

**Figure 5:**
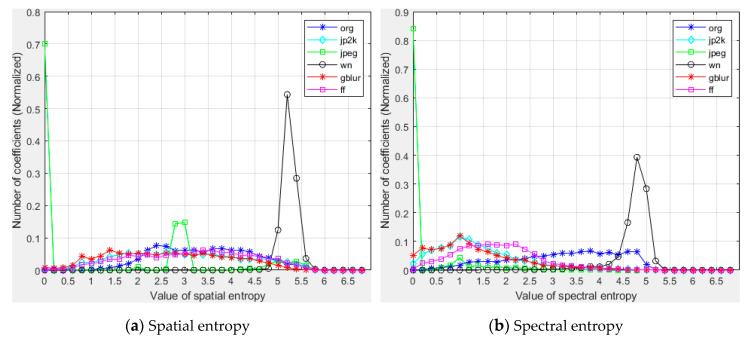
Histograms of spatial and spectral entropy values for different types of distortion. The ordinate represents the coefficient normalized between 0 and 1.

To illustrate the behavior of the local spatial entropy and spectral entropy against different degrees and types of distortions, we conducted a series of validation experiments on images. As shown in Figure 5, the undistorted image (org) has a spatial entropy histogram that is left-skewed. The spectral entropy histogram has a similar distribution. we can find different types of distortions (jp2k and jpeg compression, noise, blur and fast-fading) that exert systematically different influences on the local spatial and spectral entropy. Therefore, we utilize skewness and mean as features to measure the image quality.

#### 2.1.4. AGGD Fitting Parameter of Curvelet Coefficients

The Curvelet transform is a higher dimensional generalization of the Wavelet transform designed to represent images at different scales and different angles [26]. Therefore, it is characterized by the ability to capture the information along the edges of the image well.

Taking $f[t_{1},t_{2}](0\leq t_{1},t_{2}<n)$ as the input in Cartesian coordinate system, the discrete curvelet transform of a 2-D function $f[t_{1},t_{2}]$ is computed by:

(19) $$C(j,l,k)=\sum_{0\leq t_{1},t_{2}<n}f[t_{1},t_{2}]\overset{\_\_\_\_\_\_\_\_\_\_\_\_\_}{\varphi_{j,l,k}[t_{1},t_{2}]}$$

where $\varphi_{j,l,k}$ represents a curvelet of scale j at position index k, with angle index l, $t_{1},\;t_{2}$ denoting coordinates in the spatial domain [34].

**Figure 6:**
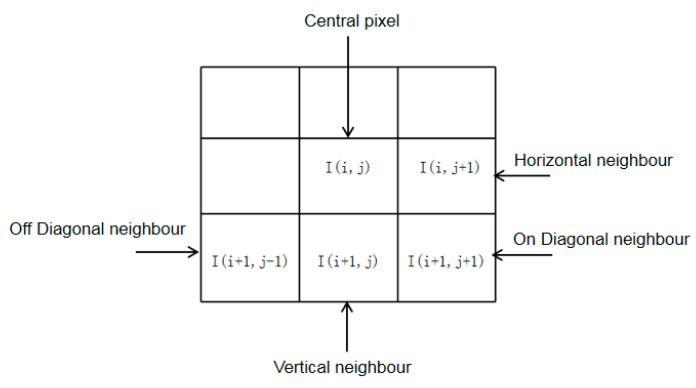
Various paired products computed in order to quantify neighboring statistical relationships. Pairwise products are computed along four orientations—horizontal, vertical, main-diagonal, and secondary-diagonal at a distance of 1 pixel.

After the curvelet transform is implemented on the distorted image, we can obtain the curvelet coefficients. Then we can compute the MSCN coefficients from the curvelet coefficient according to Equation (10) described in Section 2.1.2. While MSCN coefficients are definitely more homogenous for pristine images, the signs of adjacent coefficients also exhibit a regular structure, which gets disturbed in the presence of distortion. We construct this structure using the empirical distributions of pairwise products of neighboring MSCN coefficients along four orientations: horizontal (H), vertical (V), main-diagonal (D1), and secondary-diagonal (D2), as depicted in Figure 6, respectively.

Mathematically, it could be computed by:

(20) $$O_{b}(i,j)=M_{I}(i,j)\times M_{I}(i+1,j+b)$$

(21) $$H(i,j)=M_{I}(i,j)\times M_{I}(i,j+1)$$

where *M_I_* is the curvelet coefficients and *O_b_* represents the pairwise product of the MSCN coefficients and the MSCN coefficients in the V, D1 and D2, and *b* is set to 0, 1, −1. *H* represents the pairwise product of the MSCN coefficients and the horizontal MSCN coefficients.

**Figure 7:**
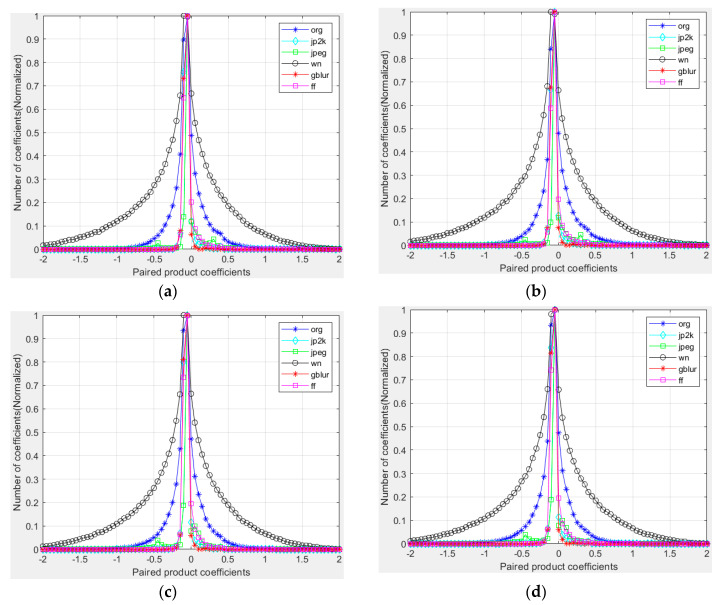
Histograms of paired products of MSCN coefficients of a natural undistorted image and various distorted versions of it. (**a**) Horizontal; (**b**) Vertical; (**c**) Main-diagonal; (**d**) Secondary-diagonal. Distortions from the LIVE IQA database. jp2k: JPEG2000. jpeg: JPEG compression. wn: additive white Gaussian noise. gblur: Gaussian blur. ff: Rayleigh fast-fading channel simulation.

In order to visualize how paired products vary in the presence of distortion, in Figure 7, we plot histograms of paired products along each of the four orientations, for a reference image and for distorted versions of it. Figure 7a–d are the histograms of the pairwise product of the center pixel and horizontal, vertical, main diagonal, and secondary-diagonal MSCN coefficients.

We use the zero mean asymmetric generalized Gaussian distribution (AGGD model) to fit its statistical distribution. The histograms of the pairwise products in four directions are calculated.

(22) $$f(x;\alpha ,\sigma_{l}^{2},\sigma_{r}^{2})=\left\{ \begin{array}{l} \frac{\alpha }{\left(\beta_{l}+\beta_{r})\Gamma (\frac{1}{\alpha }\right)}\exp(-{\left(\frac{-x}{\beta_{l}}\right)}^{\alpha })x<0\\ \frac{\alpha }{\left(\beta_{l}+\beta_{r})\Gamma (\frac{1}{\alpha }\right)}\exp(-{\left(\frac{-x}{\beta_{r}}\right)}^{\alpha })x\geq 0 \end{array}\right.$$

where

(23) $$\beta_{l}=\sigma_{l}\sqrt{\frac{\Gamma (\frac{1}{\alpha })}{\Gamma (\frac{3}{\alpha })}}$$

(24) $$\beta_{r}=\sigma_{r}\sqrt{\frac{\Gamma (\frac{1}{\alpha })}{\Gamma (\frac{3}{\alpha })}}$$

where α is the shape parameter controlling the statistical distribution, and $\beta_{l}$ and $\beta_{r}$ are the scale parameters of left and right edges respectively. When $\sigma_{l}=\sigma_{r}$, AGGD model can be transformed into generalized Gaussian model (GGD). In addition, we use the three parameters mentioned above to calculate η as an additional feature. See the formula below for the specific calculation process.

(25) $$\eta =(\beta_{r}-\beta_{l})\frac{\Gamma (\frac{2}{\alpha })}{\Gamma (\frac{1}{\alpha })}$$

Finally, the AGGD fitting parameters of curvelet coefficients are extracted *f_AGGD_* = {$\alpha ,\beta_{l},\beta_{r},\eta$}.

#### 2.1.5. Oriented Energy Distribution (OED)

Cortical neurons are highly sensitive to orientation energy in images, whereas image distortion can modify the orientation energy distribution in an unnatural manner. The curvelet transform is a rich source of orientation information on images and their distortions [26]. In order to describe changes in the energy distribution in curvelet domain, we utilize the mean of the logarithm of the magnitude of the curvelet coefficients in all scales as an energy measure to calculate the energy differences between the adjacent layers and interval layers. The energy statistical function $e_{j}$ on different scales j is calculated by:

(26) $$e_{j}=E(\log_{10}|\theta_{j}|),\;j=1,2,3,4,5$$

(27) $$\left\{ \begin{array}{l} d_{1}=e_{5}-e_{4}\\ d_{2}=e_{4}-e_{3}\\ d_{3}=e_{3}-e_{2}\\ d_{4}=e_{2}-e_{1}\\ d_{5}=e_{5}-e_{3}\\ d_{6}=e_{4}-e_{2} \end{array}\right.$$

where $\theta_{j}$ is a set of coefficients of the scale matrix’s set with scale index j, and $d_{1},d_{2},\ldots ,d_{6}$ represent energy differences between the adjacent layers and interval layers.

At the same time, the curvelet transform has rich directional information on the reference and distorted images. The magnitude of oriented energy is different in various categories of distortion. The average kurtosis *m* can be selected as the quality feature.

Previous studies [35,36,37,38,39] have found that image distortion processes affect image anisotropy. To capture this, we calculate the variation of the non-cardinal orientation energies $c_{v}$ [40]:

(28) $$c_{v}=\frac{\sigma_{\mathrm{so}}}{\mu_{\mathrm{so}}}$$

where $\mu_{\mathrm{so}}$ and $\sigma_{\mathrm{so}}$ are the sample mean and standard deviation of the non-cardinal orientation energies, and $c_{v}$ is employed to capture the degree of anisotropy of the image, and is used as a quality feature. Thus, we obtain an eight-dimensional feature group, which describes the oriented energy distribution, referred to as *f_OED_ =* [*m*,$c_{v}$,$d_{1}$,$d_{2}$,$d_{3}$,$d_{4}$,$d_{5}$,$d_{6}$].

### 2.2. Pooling Strategy

The features extracted from the dual-domain are fused to form a multi-dimensional feature vector [41,42,43,44,45,46]. After feature extraction, the quality regression from feature space to image quality is conducted, which can be denoted as

(29) $$Q=f_{Q}(F_{f})$$

where $f_{Q}(•)$ is a quality regression function achieved by feature pooling strategy, $F_{f}$ represents the extracted feature vector, and *Q* is the quality of tested image.

At present, learning-based methods [25,26,30] have been widely used in the feature pooling stage of IQA, such as support vector regression (SVR), random forest (RF) and BP neural network. SVR model is relatively fast in the regression processing, however, it is prone to over-fitting. BP neural network is employed rarely because of its high complexity. In the learning process of RF, a large number of decision trees will be generated. Each decision tree will give its own classification results, and the final regression score will be obtained by averaging the classification results of all decision trees. Many studies have shown that RF has higher prediction accuracy and is less prone to over-fitting [47,48,49], which is better than SVR in predicting the color images. Therefore, RF is used in this paper to learn the mapping relationship between feature vectors and Mean Opinion Score (MOS), so as to obtain the final quality score.

## 3. Experimental Results and Analysis

### 3.1. Database and Evaluation Criterion

**Figure 8:**
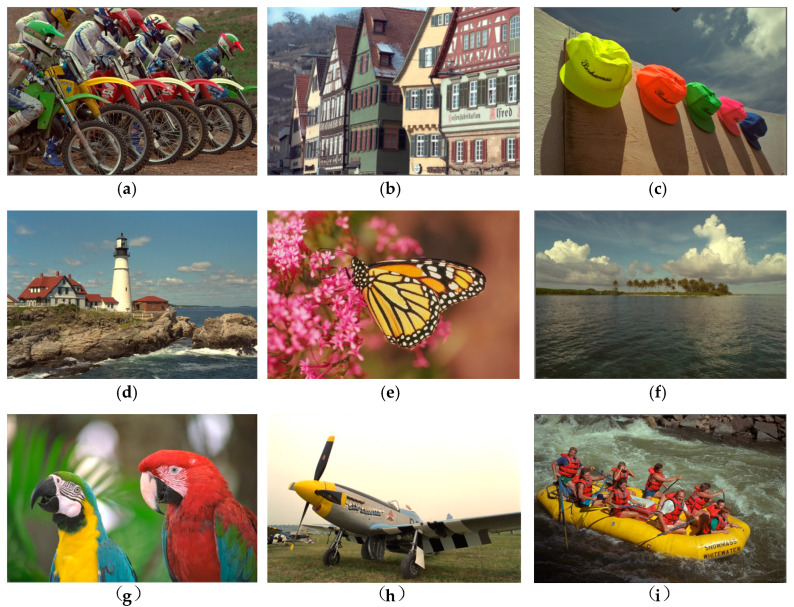
Some examples of reference scenes in the LIVE database. (**a**–**i**) shows some examples of reference scenes in the LIVE database, including “bikes scene”, “buildings scene”, “caps scene”, “lighthouse2 scene”, “monarch scene”, “ocean scene”, “parrots scene”, “plane scene” and “rapids scene” (not listed one by one due to layout reasons).

In order to verify the effectiveness of the proposed algorithm, we tested the performance of DFF-IQA on the LIVE IQA database [50], which contains 29 reference images distorted by the five distortion types: white noise, JPEG and JP2K compression, Gaussian blur, and fast Rayleigh fading, yielding 799 distorted images. Each distorted image is provided with a Difference Mean Opinion Score (DMOS) value, which is representative of the human subjective score of the image. The subjective score DMOS value range is 0–100; the larger the DMOS value is, the more serious the image distortion is. Some examples of reference scenes in the LIVE database are showed in Figure 8.

Pearson linear correlation coefficient (PLCC), Spearman rank order correlation coefficient (SROCC) and root mean square error (RMSE) were used to measure the correlation between a set of predicted visual quality scores $Q_{pre}$ and a set of predicted visual quality score $Q_{sub}$. The better correlation with human perception means a value close to 0 for RMSE and a value close to 1 for PLCC and SROCC. The calculation processes of PLCC, SROCC and RMSE are shown in Equations (30)–(32), respectively.

(30) $$PLCC(Q_{pre},Q_{sub})=\frac{\mathrm{cov}(Q_{sub},Q_{pre})}{\sigma (Q_{sub})\sigma (Q_{pre})}$$

(31) $$SROCC(Q_{pre},Q_{sub})=1-\frac{6\sum d_{i}{}^{2}}{N(N^{2}-1)}$$

where cov(⋅) represents the covariance between $Q_{pre}$ and $Q_{sub}$; σ(⋅) represents the standard deviation;$d_{i}$ is rank difference of i-th evaluation sample in $Q_{pre}$ and $Q_{sub}$; and *N* is the number of samples.

(32) $$RMSE=\sqrt{\frac{1}{N}\sum_{i=1}^{n}{\left(x_{i}-y_{i}\right)}^{2}}$$

where *N* is the number of samples; $x_{i}$ is the subjective value (MOS/DMOS); and $y_{i}$ is the predicted value by IQA model.

### 3.2. Performance Analysis of Different Features

Overall, the proposed method extracts five types of quality perception features from the distorted image, as tabulated in Table 1.

Table 2 show the performance comparison of different features on the specific distortion types of LIVE database. The overall performance of the combination of the five features is better than that of each single feature, which shows that the design of each feature is reasonable and complementary.

### 3.3. Overall Performance Analysis

We compared the performance of the proposed algorithm (DFF-IQA) with three FR-IQA models (PSNR, SSIM [4] and VIF [7]) and another five NR-IQA algorithms (BIQI [23], DIIVINE [24], BLIINDS-II [28], BRISQUE [30] and SSEQ [25]) on individual distortion types over the LIVE database. To make a fair comparison, we performed a similar random 20% test set selection for 1000 times to get median performance indices of the FR algorithms, since the FR algorithms do not need training. In addition, we only tested the FR approaches on the distorted images (excluding the reference images of the LIVE IQA database). For the NR approaches, the same random 80–20% train test trails were conducted and the median performance was treated as the overall performance indices. We also calculated the standard deviations (STD) of the performance indices to judge the algorithm stability in Table 6. Higher PLCC and SROCC with the lower STD and RMSE mean excellent quality prediction performance. The results are shown in Table 3, Table 4, Table 5 and Table 6.

Table 3, Table 4 and Table 5 are the experimental results of SROCC, PLCC and RMSE respectively. It can be seen from the results in the table that when evaluating the whole LIVE database, the median value of SROCC is 0.9576, and the median value of PLCC is 0.9671, all of which are the best performance. We also calculated the standard deviations (STD) of the performance indices to measure the stability of the models in Table 6; the proposed model also has the best stability. In addition, we can also find that the VIF model performs better than the proposed method in the median value of RMSE. However, its application is limited because it is a FR-IQA model. In summary, the proposed DFF-IQA model is more consistent with the human visual system than other competing IQA methods considering the practical application.

To further testify the superiority of the proposed DFF-IQA method, we also conducted a statistical significance analysis by following the approach in [16]. The comparison results among nine metrics are shown in Table 7 in terms of the Median PLCC. The proposed method is obviously superior to other competing IQA models, which is consistent with the data in Table 4.

**Figure 9:**
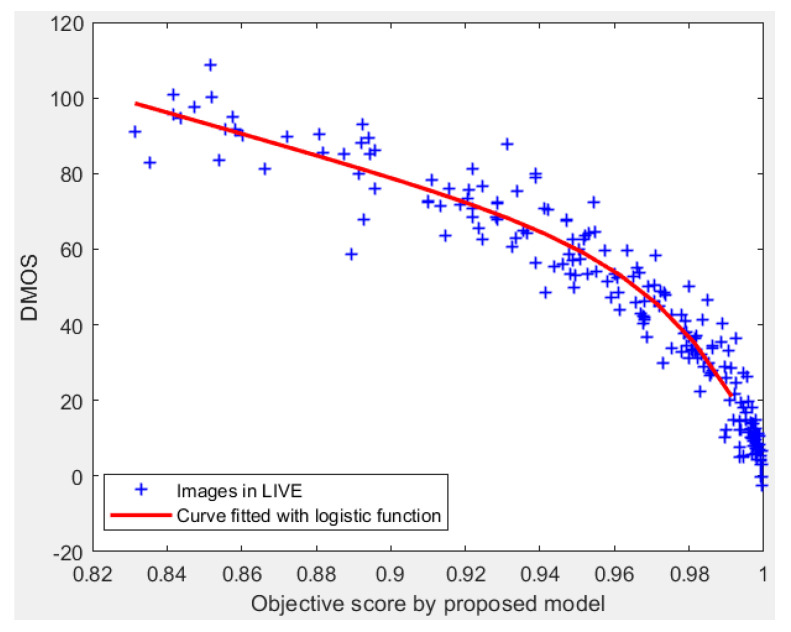
Scatter plot of the proposed model on LIVE database. Each point (‘+’) represents one test image. The red curve shown in Figure 9 is obtained by a logistic function.

In order to further analyze the prediction performance of the proposed model, we provide a visual illustration by scatter plot of subjective ratings (DMOS) versus objective scores obtained by DFF-IQA model on LIVE database. As shown in Figure 9, each point (‘+’) represents one test image. The red curve shown in Figure 9 is obtained by a logistic function. DFF’s points are more close to each other, which means that the model correlates well with subjective ratings.

**Figure 10:**
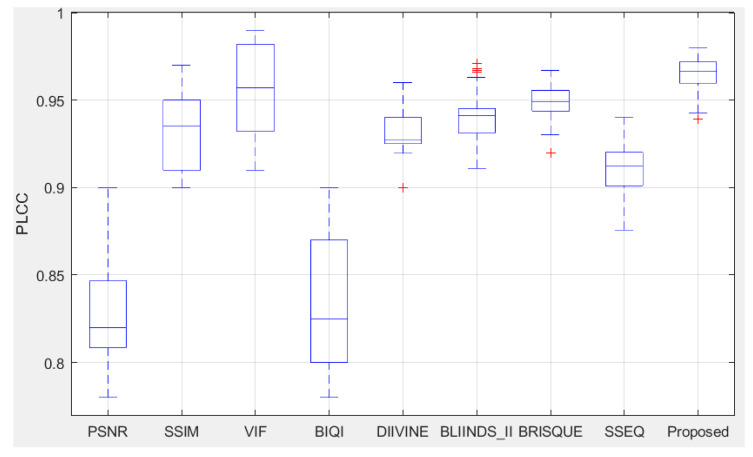
Box plot of PLCC distributions of the compared IQA methods over 1000 trials on the LIVE database.

From Figure 10, we can find the proposed method is superior to other competing FR-IQA and NR-IQA models, which is consistent with the median PLCC in Table 4. From the above box plot, we can also find that the VIF model performs as well as the proposed method. However, its application is limited because it is a full-reference method. Therefore, experimental results further confirm that the proposed FFD-IQA model has good performance and practical application.

## 4. Conclusions

In this paper, we proposed a novel metric for NR-IQA, dubbed as DFF-IQA. It is based on dual-domain features extracted. The basic consideration is to develop some known facts of the human visual system (HVS) to build an IQA model that is useful for blind quality evaluation of color images. For this purpose, the proposed DFF-IQA model is dedicated to characterizing the image quality from both spatial and frequency domains. In the spatial domain, features of weighted local binary model (WLBP), naturalness and spatial entropy are extracted. In the frequency domain, the features of spectral entropy, asymmetrical generalized gaussian distribution (AGGD) fitting parameters and oriented energy distribution (OED) of curvelet coefficient are extracted. Then, the features extracted in the dual domain are fused to form a feature vector. At last, random forest (RF) is adopted to build the relationship between image features and quality scores, yielding a measure of image quality. Experiments on LIVE databases well demonstrate the superiority of the proposed DFF-IQA model. In the future, we will consider to further improve the performance of the algorithm by extracting more effective perceptional features.

## Figures and Tables

**Table 1:** Features used for Proposed IQA method.

Feature Vector	Feature Description
*f_Naturalness_*	GGD fitting parameter describing the image naturalness
*f_OED_*	Mean kurtosis, anisotropy and scalar energy distribution
*f_Entropy_*	Means of spatial-spectral entropy and skews values for 3 scales
*f_WLBP_*	Histogram statistics of gradient weighted local binary patterns
*f_AGGD_*	AGGD parameter fitting in curvelet domain

**Table 2:** Performance comparison of different features (LIVE database).

Type	Metric	*f_Naturalness_*	*f_OED_*	*f_Entropy_*	*f_WLBP_*	*f_AGGD_*	*f_Overall_*
JP2K	PLCC	0.9007	0.9415	0.7970	0.9463	0.9211	0.9563
	SROCC	0.8830	0.9270	0.7427	0.9240	0.9030	0.9564
	RMSE	10.6202	8.2273	14.3495	7.8592	8.6202	7.0194
JPEG	PLCC	0.9183	0.9399	0.8895	0.9400	0.9311	0.9492
	SROCC	0.8898	0.9232	0.8671	0.9165	0.9098	0.9604
	RMSE	9.5544	8.1526	10.8706	8.2742	7.8544	7.5815
Noise	PLCC	0.9332	0.9807	0.9550	0.9694	0.9511	0.9938
	SROCC	0.9509	0.9805	0.9533	0.9689	0.9798	0.9895
	RMSE	7.5913	4.3086	6.4898	2.6210	5.5844	2.4098
Blur	PLCC	0.9515	0.9498	0.7235	0.9570	0.9520	0.9659
	SROCC	0.9518	0.9511	0.6786	0.9355	0.9519	0.9574
	RMSE	6.3231	6.6898	14.9504	6.2661	6.2230	5.6003
FF	PLCC	0.8424	0.8757	0.6537	0.9181	0.8920	0.9214
	SROCC	0.8185	0.8509	0.5613	0.8878	0.8519	0.9080
	RMSE	11.8343	10.4614	14.3495	8.8186	9.2230	8.4213

**Table 3:** Median SROCC across 1000 train-test trials on the LIVE IQA database. From the indices, we can see that the proposed approach shows the best performance in the individual distortion types (JP2K, JPEG and Noise) and in all distorted types.

Model	JP2K	JPEG	Noise	Blur	FF	All
PSNR	0.8991	0.8483	0.9834	0.8078	0.8985	0.8294
SSIM	0.9512	0.9174	0.9696	0.9514	0.9553	0.8995
VIF	0.9514	0.9105	0.9845	0.9723	0.9632	0.9522
BIQI	0.8552	0.7766	0.9765	0.9257	0.7696	0.7598
DIIVINE	0.9353	0.8922	0.9827	0.9552	0.9097	0.9175
BLIINDS-II	0.9463	0.9351	0.9635	0.9335	0.8993	0.9332
BRISQUE	0.9458	0.9252	0.9893	0.9512	0.9027	0.9297
SSEQ	0.9422	0.9512	0.9785	0.9484	0.9036	0.8753
Proposed	0.9564	0.9604	0.9895	0.9574	0.9080	0.9576

**Table 4:** Median PLCC across 1000 train-test trials on the LIVE IQA database. From the results, we can find that the VIF model indicates best performance in the individual distortion types of JP2K, Blur and FF. However, the proposed method shows the best index in the whole LIVE database.

Model	JP2K	JPEG	Noise	Blur	FF	All
PSNR	0.8836	0.8514	0.9816	0.8007	0.8938	0.8082
SSIM	0.9602	0.9486	0.9862	0.9538	0.9618	0.9102
VIF	0.9665	0.9479	0.9925	0.9775	0.9697	0.9522
BIQI	0.8415	0.7605	0.9733	0.9117	0.7343	0.7423
DIIVINE	0.9410	0.9098	0.9745	0.9394	0.9127	0.9117
BLIINDS-II	0.9494	0.9506	0.9615	0.9374	0.9080	0.9242
BRISQUE	0.9473	0.9331	0.9884	0.9465	0.9143	0.9494
SSEQ	0.9465	0.9703	0.9807	0.9608	0.9199	0.9126
Proposed	0.9563	0.9492	0.9938	0.9659	0.9214	0.9671

**Table 5:** Median RMSE across 1000 train-test trials on the LIVE IQA database. From the experimental data, we can deduce the proposed method shows second performance in the whole database. The best IQA model is the VIF.

Model	JP2K	JPEG	Noise	Blur	FF	All
PSNR	7.5642	8.3268	3.0743	9.4292	7.3991	9.4974
SSIM	4.5392	5.0772	2.6585	4.6825	4.4856	6.6356
VIF	4.1945	5.0855	1.9606	3.3313	3.9622	4.9182
BIQI	13.7872	17.0135	5.3805	9.6563	15.5516	15.9546
DIIVINE	8.5705	10.6071	5.2138	8.0665	9.6522	9.9346
BLIINDS-II	8.1732	7.7657	6.5012	8.0698	9.7143	9.0475
BRISQUE	8.3627	9.3784	3.5295	7.5637	9.4362	7.2741
SSEQ	7.8286	5.8468	4.3213	6.0029	8.5420	9.3971
Proposed	7.0194	7.5815	2.4098	5.6003	8.4213	5.8249

**Table 6:** Standard deviation of SROCC, PLCC and RMSE across 1000 train-test trials on the LIVE database. From the results, we can find the proposed method shows best performance in PLCC STD, second in SROCC STD, and third in RMSE STD.

Model	PLCC STD	SROCC STD	RMSE STD
PSNR	0.0250	0.0567	2.5851
SSIM	0.0097	0.0145	0.4736
VIF	0.0068	0.0073	0.4324
BIQI	0.0655	0.0664	1.5995
DIIVINE	0.0274	0.0287	1.2706
BLIINDS-II	0.0236	0.0248	1.1654
BRISQUE	0.0118	0.0141	0.9456
SSEQ	0.0174	0.0198	1.1651
Proposed	0.0067	0.0078	0.5733

**Table 7:** Statistical significance tests of different IQA models in terms of PLCC. A value of ‘1’ (highlighted in green) indicates that the model in the row is significantly better than the model in the column, while a value of ‘0’ (highlighted in purple) indicates that the model in the row is not significantly better than the model in the column. The symbol “--” (highlighted in blue) indicates that the models in the rows and columns are statistically indistinguishable.

Model	PSNR	SSIM	VIF	BIQI	DIIVINE	BLIINDS-II	BRISQUE	SSEQ	Proposed
**PSNR**	--	0	0	1	0	0	0	0	0
**SSIM**	1	--	0	1	0	0	0	0	0
**VIF**	1	1	--	1	1	1	1	1	0
**BIQI**	0	0	0	--	0	0	0	0	0
**DIIVINE**	1	1	0	1	--	0	0	0	0
**BLIINDS-II**	1	1	0	1	1	--	0	0	0
**BRISQUE**	0	0	1	0	0	0	--	0	0
**SSEQ**	1	1	0	1	1	1	1	--	0
**Proposed**	1	1	1	1	1	1	1	1	--
